# Increased expression of metabolism and lysosome-associated genes in a *C. elegans* *dpy-7* cuticle furrow mutant

**DOI:** 10.17912/micropub.biology.001241

**Published:** 2024-07-31

**Authors:** Aiden Fong, Michael Rodriguez, Keith Patrick Choe

**Affiliations:** 1 Biology, University of Florida, Gainesville, Florida, United States; 2 Department of Biology and Genetics Institute, University of Florida, Gainesville, FL USA

## Abstract

The collagen-based epidermal ‘cuticle' of *Caenorhabditis elegans* functions as an extracellular sensor for damage that regulates genes promoting osmotic balance, innate immunity, and detoxification. Prior studies demonstrate that SKN-1 , an ortholog of the mammalian Nrf transcription factors, activates core detoxification genes downstream from cuticle damage. Prior RNAseq data suggested that expression of five genes with functions in redox balance, ATP homeostasis, and lysosome function ( *gst-15* , *gst-24* , *cyts-1* , *argk-1* , and *mfsd-8.4* ) were increased in a cuticle collagen mutant; this study employed RT-qPCR to verify this observation and to test the role of SKN-1 . Activation of all five genes was verified in *dpy-7* mutants, but none were reduced by *skn-1 (RNAi)* suggesting parallel or distinct regulatory mechanisms.

**Figure 1. Expression data in wild type and dpy-7(e88) mutant worms with and without skn-1(RNAi):**
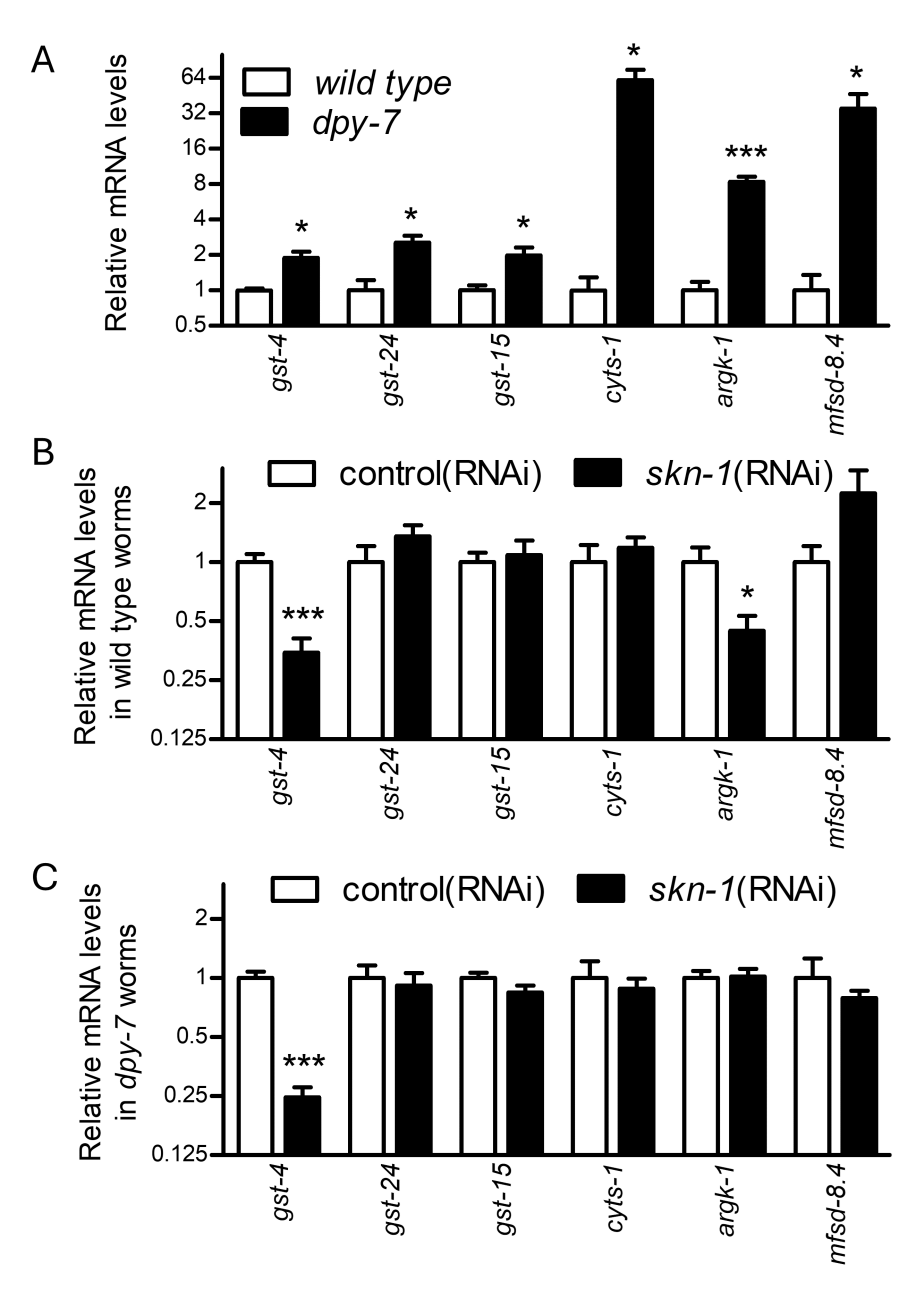
Relative mRNA expression levels of genes in wild type vs *dpy-7 ( e88 )* mutant worms (A) and the effects of *skn-1* RNAi in wild type (B) and *dpy-7* worms (C). *P < 0.05 or ***P < 0.001, normalized by *rpl-2* and compared to expression levels in controls. N = 5 or 10 replicate cDNA samples from 10 L4 larval worms each.

## Description

Animal cells rely on conserved signaling mechanisms to sense adverse environmental conditions and modulate expression of cytoprotective genes. Intracellular sensing and signaling pathways that regulate cytoprotective genes are well-studied (Choe et al. 2009, Blackwell et al. 2015, Dietrich et al. 2017, Urso and Lamitina 2021, Pujol and Ewbank 2022) , but mechanisms outside of cells in the tissues that interact directly with the environment are poorly understood.

Collagenous extracellular matrices (ECMs) are ubiquitous in animal organs and serve as barriers to the environment in epidermal tissues. Although originally hypothesized to be inert physical scaffolds, ECMs are now understood to be dynamic structures that regulate organogenesis and tissue remodeling (Maquart et al. 2004, Rozario and DeSimone 2010, Clause and Barker 2013) . In mammalian lungs, peptide fragments of digested collagen and other ECM components are sensed by cell receptors and regulate immune responses, wound repair, and cell proliferation (Gaggar and Weathington 2016, Patel and Snelgrove 2018) .

Nematodes are enclosed in a collagen-rich exoskeleton called the ‘cuticle' (Chisholm and Hsiao 2012) . We and others have identified the cuticle as a putative extracellular sensor for damage that regulates three stress responses (Lamitina et al. 2006, Wheeler and Thomas 2006, Pujol et al. 2008, Dodd et al. 2018, Wimberly and Choe 2022) . This ECM damage response is induced by disruption of circumferential bands of collagen in the cuticle known as annular furrows; silencing or mutation of any one of six collagens required for furrow formation activates the responses (Dodd et al. 2018) . Understanding this ECM damage response will help define a novel mode of stress-response signaling and relevant homeostasis mechanisms. The mechanism for sensing cuticle damage is not known, but recent studies provide insights. Full activation of stress responses requires atypical membrane-associated kinase DRL-1 (Wimberly and Choe 2022) . Plasma membrane folds named ‘meisosomes' were recently identified and shown to be associated with furrows in epidermal cells and could be involved in signaling (Aggad et al. 2023) .

Candidate genes activated by furrow disruption have been identified with microarrays and RNAseq; they are highly enriched for functions in canonical osmotic, detoxification, and innate immune responses and largely exclude other core stress responses (Pujol et al. 2008, Rohlfing et al. 2010, Dodd et al. 2018, Scolaro et al. 2019) . DPY-7 is a collagen localized to furrows and is required for periodic organization of the cuticle and epidermal cortical cytoskeleton and attachment of cuticle to the epidermal plasma membrane (Cox et al. 1980, McMahon et al. 2003, Thein et al. 2003, Dodd et al. 2018, Chandler and Choe 2022, Aggad et al. 2023) . In the current study, we used RT-qPCR to independently verify activation of genes predicted to function in detoxification, redox balance, and energy metabolism in *dpy-7 ( e88 )* mutant worms; we also used RNAi to test the requirement of transcription factor SKN-1 , a master regulator of detoxification that we previously showed to mediate activation of *gst-4* and *gst-10* in the same strain (Dodd et al. 2018) . Sequencing of the *skn-1* ORFeome clone that we used confirmed that it covers exons 1-4 of *skn-1 c* , which overlaps at least 227 bases of all predicted transcript variants (i.e., *skn-1 a, b, c,* and *d)* .

As shown in Figure 1A, *gst-15* , *gst-24* , *cyts-1* , *argk-1* , and *mfsd-8.4* were all verified to be induced in *dpy-7* worms; direct SKN-1 target gene *gst-4* was previously studied and is included here as a positive control (Dodd et al. 2018) . *cyts-1* is predicted to encode a cysteine synthase and was induced 61.2-fold; cysteine is a precursor for glutathione, a major cellular redox buffer (Lapenna 2023) . *gst-15* and *gst-24* are predicted to encode glutathione S-transferase enzymes and they were induced 1.8-2.5-fold; glutathione S-transferases conjugate glutathione to small molecules reducing toxicity and increasing solubility (Salinas and Wong 1999) . Activation of these detoxification and redox homeostasis genes is expected to help compensate for a compromised barrier ECM that is permeable to xenobiotics (Dodd et al. 2018) . Surprisingly, only expression of positive control gene *gst-4* was reduced by *skn-1* RNAi in wild type and *dpy-7* worms (Figures 1B-C).

*argk-1* is predicted to encode a creatine kinase and was induced 8.3-fold in *dpy-7* worms ( Figure 1A ); creatine kinases function to buffer and transport energy and are enriched in muscle and neurons (Sumien et al. 2018) . In human cells and aquaculture turtles, infection has been linked to upregulation of creatine kinase expression, potentially functioning to buffer ATP demands in tissues mounting immune-responses (Li et al. 2020) . Single cell expression data suggest that *argk-1* is expressed in the hypodermis and intestine (Paker 2019). Worms with disrupted furrows synthesize high levels of the energetically expensive osmolyte glycerol in these same tissues (Lamitina et al. 2006, Possik et al. 2015, Dodd et al. 2018) ; activation of *argk-1* could function to buffer ATP levels under these conditions. Basal expression of *argk-1* was reduced by *skn-1* RNAi, but not in *dpy-7* worms (Figures 1B-C).

*mfsd-8.4* encodes a homolog of lysosomal chloride ion membrane transporter MFSD8 (Wang et al. 2021) and was induced 35.1-fold in *dpy-7* worms. MFSD8 function and regulation are poorly understood; MFSD8 mutations are associated with neuronal ceroid lipofuscinoses disease in humans and with defects in protein secretion and lysosomal function in amoeba (Kirola et al. 2022, Yap et al. 2022) . Single cell expression data suggest that *mfsd-8.4* is expressed in interneurons under basal conditions (Paker 2019). Lysosomes are remodeled during molting and impairing lysosome function causes molting defects (Miao et al. 2020) . If *mfsd-8.4* is expressed in epidermal cells of *dpy-7* worms, it could function to promote digestion of damaged cell components or secretion of proteins involved in regulation of cuticle remodeling. Expression of *mfsd-8.4* was not reduced by *skn-1* RNAi (Figures 1B-C).

Our results expand the diversity of genes activated by the cuticle damage response to include cysteine synthesis, energy metabolism, and lysosomal function. Unlike *gst-4* and some other detoxification genes (Dodd et al. 2018) , none of these newly verified responses to *dpy-7* mutation were dependent on *skn-1* . There could be redundant or distinct mechanisms of activation; future studies could test the role of transcription factors ELT-3 and STA-2 that we and others previously showed to mediate parts of the response to *dpy-7* mutation (Zugasti et al. 2014, Dodd et al. 2018). Creatine kinases and MFSD8 play important roles in human physiology and pathophysiology. Strong activation in *dpy-7* worms provides a model for understanding regulation and function in the context of stress response.

## Methods

Worms were maintained on OP50 *E. coli* on NGM agar at 20°C with standard conditions. For experiments, worm eggs were released with bleach and raised on dsRNA-expressing *E. coli* ( HT115 (DE3)); clone pPD129.36 (LH4440) encoding a 202-bp dsRNA not homologous to *C. elegans* genes was used as a control and the *skn-1* dsRNA clone was derived from the ORFeome library (Open Biosystems, Huntsville, AL) as we have described previously (Choe et al. 2009, Tang and Choe 2015) .

Worms were collected and processed for RT-qPCR at the L4 stage (to avoid embryos) as we have described previously (Scolaro et al. 2019, Piloto et al. 2022) with slight modifications. After lysis, gDNA was degraded using DNase (Thermo Fisher EN007). Primers were designed using Primer-BLAST (U.S. National Library of Medicine) and span intron splice junctions. mRNA levels were normalized to *rpl-2* and to controls using the delta-delta Ct method. Statistical significance was analyzed with Students t-tests and P-values and were corrected for multiple comparisons with Benjamini-Hochberg adjustments.

## Reagents

Strains:

*C. elegans* strains used were wild-type N2 Bristol and CB88 *dpy-7 ( e88 )* , which are both available at the *Caenorhabditis* Genetics Center.

Primers:

*rpl-2* – CTTTCCGCGACCCATACAA and CACGATGTTTCCGATTTGGAT

*gst-4 ­* – TCCGTCAATTCACTTCTTCCG and AAGAAATCATCACGGGCTGG

*gst-24* – GGAGCGTTGAAGCCAAAAAC and TTGGGGGATTTCGAAGCCAT

*gst-15 ­* – AGAAAATGAGAGACAAAACCCCA and AGATTGGGGGATGTCGAAGC

*cyts-1* – TTCGCCGTAGTTTCTGAGGA and CGGAGAGCAGTTGGTACCTTTAT

*argk-1* – CTGCGATAAGCTTGACCTCCA and TCCGAGACGAGCCCTGTTA

*mfsd-8.4* – CCAGACAAGACAGGAAGCAGT and AGAATCGTGGCAATGAATCCAG

RNAi:

HT115 *E. coli* with empty plasmid pPD129.36 (LH4440) or with the ORFeome *skn-1* clone that covers *skn-1 c* exons 1-4 and overlaps with all predicted transcript variants (i.e., *skn-1 a-d)*
